# Retinal transcriptome sequencing sheds light on the adaptation to nocturnal and diurnal lifestyles in raptors

**DOI:** 10.1038/srep33578

**Published:** 2016-09-20

**Authors:** Yonghua Wu, Elizabeth A. Hadly, Wenjia Teng, Yuyang Hao, Wei Liang, Yu Liu, Haitao Wang

**Affiliations:** 1 Jilin Provincial Engineering Laboratory of Avian Ecology and Conservation Genetics, Northeast Normal University, 5268 Renmin Street, Changchun, 130024, China; 2 Department of Biology, Stanford University, 371 Serra Mall, Stanford, CA 94305-5020, USA; 3 Biomarker Technologies Corporation, Beijing 101300, China; 4 Jilin Provincial Key Laboratory of Animal Resource Conservation and Utilization, Northeast Normal University, 2555 Jingyue Street, Changchun 130117, China; 5 Key Laboratory of Vegetation Ecology, Ministry of Education, Institute of Grassland Science, Northeast Normal University, 5268 Renmin Street, Changchun, 130024, China; 6 Ministry of Education Key Laboratory for Tropical Animal and Plant Ecology, College of Life Sciences, Hainan Normal University, Haikou 571158, China

## Abstract

Owls (Strigiformes) represent a fascinating group of birds that are the ecological night-time counterparts to diurnal raptors (Accipitriformes). The nocturnality of owls, unusual within birds, has favored an exceptional visual system that is highly tuned for hunting at night, yet the molecular basis for this adaptation is lacking. Here, using a comparative evolutionary analysis of 120 vision genes obtained by retinal transcriptome sequencing, we found strong positive selection for low-light vision genes in owls, which contributes to their remarkable nocturnal vision. Not surprisingly, we detected gene loss of the violet/ultraviolet-sensitive opsin (*SWS1*) in all owls we studied, but two other color vision genes, the red-sensitive *LWS* and the blue-sensitive *SWS2*, were found to be under strong positive selection, which may be linked to the spectral tunings of these genes toward maximizing photon absorption in crepuscular conditions. We also detected the only other positively selected genes associated with motion detection in falcons and positively selected genes associated with bright-light vision and eye protection in other diurnal raptors (Accipitriformes). Our results suggest the adaptive evolution of vision genes reflect differentiated activity time and distinct hunting behaviors.

After the K-T mass extinction, birds experienced an explosive radiation, diversifying and adapting to many different terrestrial and aquatic niches from the tropics to the poles^1^,^2^. Although birds are primarily diurnal, one important exception is owls (Strigiformes), which are typically crepuscular or nocturnal. The earliest fossil record of owls goes back into the Paleocene, with a subsequent adaptive radiation during the Eocene and Oligocene^3^, a time when rodents, a primary food source for owls, also diversified^4^. In order to adapt to dim-light activity, the visual system of owls has undergone substantial evolutionary modification. Owls evolved large, tubular-shaped and forward-facing eyes, conferring them good stereoscopic vision, a feature common to some avian and mammalian predators^5^. Additionally, owls possess large and rod-dominant retinas, which are extremely sensitive to light, giving them extraordinary night vision^6^. Compared with owls, diurnal and/or crepuscular raptors, e.g. hawk, eagle and falcons, possess cone-dominant retinas, which confer sharp visual acuity and color perception in relatively bright-light environment^6^. Expansion into a crepuscular and/or nocturnal lifestyles is associated with functional diversification as documented by morphological, anatomical and behavioral studies^6^, but the molecular basis that underlies the diversification of the owl visual system to low-light remains unknown.

Vision in vertebrates begins with the absorption of light in the retinal photoreceptor cells (rods and cones) and activates a series of biochemical reactions known as the phototransduction cascade, which converts light into an electrical signal. Rods are specialized for vision in dim light and cones mediate vision in bright light. The phototransduction cascade process in rods and cones are similar except for their use of different isoforms^7^. The phototransduction cascade is activated by light absorption in visual pigments, which activate the G-protein (Gt). Gt, in turn, activates the cGMP phosphodiesterase (PDE6). Active PDE6 hydrolyzes cGMP to GMP, thereby lowering the intracellular concentration of cGMP and eventually resulting in closure of cGMP-gated (CNG) channels and hyperpolarizing the cell. The closure of CNG channels further leads to a decrease of cytoplasmic Ca^2+^ concentration by a Na^+^/Ca^2+^-K^+^ exchanger (NCKX), and thereby restores the cGMP concentration through activating guanylyl cyclase activating proteins (GCAPs) and hence guanylyl cyclase (GC), leading to the re-opening of CNG channels. The restoration of cGMP concentration incorporated with an efficient inactivation of each of the activated components involved in the phototransduction cascade is considered to be important for photoresponse recovery^8^. A timely photoresponse recovery in the photoreceptor is essential so that it can respond to subsequently absorbed photons, which is thought to be critical for effective temporal resolution of vision and hence motion detection^9^.

In this study, we performed a comparative evolutionary analysis of vision genes of 15 species of owls and close relatives within Afroaves (Coraciimorphae and Accipitriformes) and its sister clade, Australaves (Falconiformes) (Fig. 1) using retinal transcriptome sequencing to understand the molecular basis of the vision system in these groups. For each species, raw reads generated by transcriptome sequencing were subjected to a filtering of adaptors and low-quality reads, and only high-quality reads were used for *de novo* assembly using Trinity software^10^, yielding assembled unigenes for each species (Supplementary Table 1). The assembled unigenes were highly reliable with an averaged sequence identity of 99.6% compared with the sequences obtained by clonal sequencing of one selected gene (*RH1*) (Supplementary Table 2). In addition to the 15 assembled unigene sets for each of the 15 species, we included gene sequences of 11 species collected from GenBank. We then assessed adaptive evolution across a total of 120 vision genes (Supplementary Tables 3 and 4) from the 26 species. Positively selected genes (PSGs) in owls and their relatives were found to be largely different from each other (Fig. 1, Table 1), which may reflect their different activity time and behaviors. Moreover, a convergent or parallel molecular evolution of vision genes in owls and falcons was inferred.

## Results and Discussion

### Owls: Enhanced dim-light vision and reduced daytime (color) vision

Among 119 vision genes examined in owls, we found 9 genes to be under positive selection, including 5 dim-light vision genes (*PDE6B*, *CNGB1*, *CNGA1*, *SAG* and *SLC24A1*) involved in the phototransduction pathway in rods (Fig. 2). *PDE6B*, *CNGB1* and *CNGA1* play a role in activation of phototransduction, and *SAG* and *SLC24A1* play a role in photoresponse recovery or adaption. Specifically, *PDE6B* encodes the catalytic PDE-*β* subunit, which hydrolyzes cGMP into GMP. *CNGA1* and *CNGB1* respectively encode the *α* subunit and *β* subunit of CNG channels. *SAG* is known to bind to and inactivate photoactivated-phosphorylated rhodopsin (*RH1*). *SLC24A1* showed the strongest positive selection signal (ω = 999, *P* = 0.002) based on the branch model, and the ω value was also determined to be significantly larger than one (*P* = 0.047) based on the likelihood ratio test with ω fixed to one in the null model. *SLC24A1* encodes Na^+^/Ca^2+^-K^+^ exchanger, extruding free calcium in the outer segment of rods and allowing the restoration of the cGMP concentration. The positive selection observed in five dim-light vision genes involved in the activation and recovery of photoresponse may help to enhance the sensitivity to light and therefore possibly contribute to high visual acuity and high temporal resolution of vision in dim light conditions. In addition to this molecular adaptation, for visual sensitivity, the evolution of rod-dominated retina and forward-facing eyes in owls are also considered to be specialized for visual sensitivity^6^,^11^. Furthermore, owls are found to have eyes with larger corneal diameters relative to axial lengths compared to their diurnal and crepuscular counterparts, additionally optimizing them for visual sensitivity^12^,^13^. The molecular and morphological adaptations underlying visual sensitivity in owls are consistent with their behavior which demonstrates relatively high vision acuity and contrast sensitivity in scotopic conditions that is found in owls compared with other diurnal birds^14^.

We also analyzed the adaptive evolution in four opsin genes (*LWS*, *RH2*, *RH1* and *SWS2*) in owls. Intriguingly, strong signals of positive selection were detected in the red-sensitive *LWS* (long-wavelength-sensitive) gene and the blue-sensitive *SWS2* (short-wavelength-sensitive) gene along the ancestral branch of owls based on the branch-site model, and a number of positively selected sites were identified (Supplementary Fig. 1). Both of these opsin genes are known to have cone-specific expression and normally mediate daytime (color) vision. The strong positive selection of *LWS* and *SWS2* found in owls is difficult to explain since owls are known to be less active during the day. We speculate that this might be due to the ancestral niche switch of owls from diurnality to crepuscularity. Many extant owls are known to be active in crepuscular dawn and dusk hours^11^, in which the spectral composition is different from that of daytime^15^. If this is the case, the occurrences of spectral tuning of the two visual pigments under positive selection for maximizing photo capture in crepuscularity would be expected. To test this possibility, the spectral tunings of the two visual pigments were analyzed. Genetically, the spectral sensitivities of visual pigments are considered to be controlled by some critical amino acid sites, and the effects of the amino acid replacements in these sites on the wavelengths of maximal absorption (λmax) have been determined in vertebrates^16^. To detect these critical amino acid replacements in ancestral owls, we reconstructed the ancestral amino acid sequences for the two PSGs *LWS* and *SWS2* as well as *RH2* and *RH1*, respectively (Supplementary Figs 2, 4, 6 and 7), based on an empirical Bayes approach^17^. Among the four pigments analyzed, only two PSGs *LWS* and *SWS2* each harbored one critical amino acid replacement along the ancestral owl branch, in which S167A (corresponding to S164A in bovine rhodopsin) and S223A (corresponding to S292A in bovine rhodopsin) were found, respectively (Supplementary Figs 2–7). These two amino acid changes both received high posterior probability support (≥99.4%), and were considered to reduce λmax of *LWS* by 7 nm and increase λmax of *SWS2* by 8 nm, respectively (Supplementary Table 5). Moreover, no further amino acid changes in the critical amino acid sites were found in other owl branches except for the long-ear owl (*Asio otus*), in which another amino acid change T272A (corresponding to T269A in bovine rhodopsin) reducing λmax by 15 nm in *LWS* was found (Supplementary Table 5). These results suggest that all six owl species studied retained the two critical amino acid changes and thus the spectral sensitivity of *LWS* and *SWS2* might have changed little since their most recent common owl ancestor.

The short-wavelength shift of *LWS* and the long-wavelength shift of *SWS2* found in owls were compatible with the result reported in the Tawny Owl (*Strix aluco*), the only extant owl species that has had the λmax of cone visual pigments spectrophotometrically measured^18^. In the Tawny Owl, a short-wavelength shift of λmax 555 nm in *LWS* (compared with an average of 565 nm of diurnal birds) and a long-wavelength shift λmax 463 nm in *SWS2* (compared with an average of 448 nm of diurnal birds) have previously been reported^19^. The short-wavelength shift of *LWS* and the long-wavelength shift of *SWS2* in Tawny Owl seem to approach the dominant wavelengths in twilight. One previous study shows that the twilight spectral composition in forests or woodlands presents two dominant wavelengths, in which one ranges from 400 nm to around 570 nm with a λmax peak around 460 nm, and another one beginning around 630 nm and peaking around 670 nm, with a deficiency in middle wavelengths (570–630 nm)^15^. Given that cones are generally functional in a crepuscular condition but not in very dark situations (e.g. at night) mediating the so-called mesopic vision^20^, the λmax adjustment of the two cone-specific opsins *LWS* and *SWS2* in owls may likely help owls to maximize photon absorption by tuning their photoreceptor spectral sensitivities to the dominant wavelengths of light from 400 nm to around 570 nm in twilight to increase total sensitivity at relatively low light levels. This is consistent with the well-known crepuscular activities of many extant owls^11^. Moreover, our findings of the relatively strong positive selection signals of *LWS* and *SWS2* may imply an unappreciated significance of color vision during their crepuscular activities. We could not exclude the possibility of the adaptation of *LWS* and *SWS2* in owls for night color vision, as reported in hawkmoths and geckos^21^.

*SWS1* (violet-sensitive or ultraviolet (UV)-sensitive) transcripts were not found in any of the six owl species studied or the two species of Accipitriformes (the cinereous vulture (*Aegypius monachus*) and black-winged kite (*Elanus caeruleus*)), suggesting an inactive function of *SWS1* in these species. The putative loss of *SWS1* in owls is consistent with the results of previous studies indicating that UV vision is not found in the Tengmalm’s owl (*Aegolius funereus*) and UV-receptors are not found in the Tawny Owl (*Strix aluco*)^18^,^22^. The loss of *SWS1* in owls may suggest their reduced need for violet or UV vision in dim-light conditions. Mammals that live in dim environments tend to lose their *SWS1*^23^. Moreover, the green-sensitive *RH2* transcripts were not found in the grass owl (*Tyto longimembris*), consistent with the pseudogenization of *RH2* in the barn owl (*Tyto alba*)^24^. Both owl species that show loss of *RH2* belong to the family Tytonidae, suggesting even further reduction in daylight or color vision. Similarly, a more reduced visual pathway in Tytonidae has been observed when compared with another family (Strigidae) of owls^25^, possibly due to an increase in dependence on hearing in the Tytonidae. Besides the loss of two cone opsins, we were also incapable of detecting the transcripts of *GUCA1C* from the five owl species in Strigidae. *GUCA1C* is a cone-specific protein and is involved in photoresponse recovery through activating guanylyl cyclase. The loss of *GUCA1C* in Strigidae might be due to its pseudogenization as has been found in the mouse (*Mus musculus*)^26^. The possible losses of the three cone-specific genes from owls may suggest their reduced dependence on daytime (color) vision, consistent with their reduced activity in daytime.

We also found two PSGs (*ABCA4* and *PCDH15*) that seem to play an important role in maintaining normal photoreceptor function in owls. *ABCA4* is expressed in both rods and cones and is thought to prevent accumulation of the toxic substances N-retinylidene-PE generated during the phototransduction process, allowing the released all-*trans*-retinal to enter the visual cycle^27^. *PCDH15* putatively plays a role in the development and maintenance of photoreceptor cells^28^. Maintaining or strengthening the normal function of this photoreceptor would be selectively advantageous for owls since they are known to suffer from potential risk of eye injury in dim light situations^29^,^30^.

### Falcons: Molecular basis for detecting motion

Among 117 genes examined in falcons, six PSGs including *GRK1*, *ARR3*, *SLC24A1*, *GUCY2D*, *GUCY2F* and *GUCA1A* were found. Intriguingly, all the six genes are involved in photoresponse recovery or light adaption (Fig. 2). *GRK1* is rhodopsin kinase, involved in the inactivation of activated rhodopsin in rods. *ARR3* is cone-specifically expressed and is involved in inactivation of cone opsins. Behaviorally, *ARR3* deficiency is known to lead to a reduced temporal resolution of vision in zebrafish^31^. *SLC24A1* plays a crucial role in extruding free calcium in the rod outer segment and stimulating reopening of CNG channels. *GUCY2D* and *GUCY2F* encode guanylyl cyclases, which are associated with the resynthesis of cGMP, and *GUCA1A* plays a role in stimulating guanylyl cyclase to resynthesize cGMP as the lowering of the intracellular Ca^+2^ levels^32^. The roles of these six genes in the inactivation of components in the phototransduction pathway or in the restoration of cGMP are essential for photoresponse recovery or light adaption and therefore may contribute to a high temporal visual resolution, which is thought to be crucial for detecting motion^9^.

Indeed, falcons are characterized by their high-speed flight for catching birds and insects in the air^33^,^34^ and have remarkable visual acuity for detecting movement^34^. During the high-speed pursuit of prey, falcons are likely to encounter rapid differences in light, and a fast temporal resolution of vision would be advantageous to detect fast-flying prey. Moreover, the six genes under positive selection are known to be involved in the phototransduction pathway of either cones or rods or both, which suggests that falcons have a high temporal resolution of vision in both bright-light and dim-light conditions. In particular, we found falcons harbored one positively selected dim-light vision gene, *SLC24A1*, with the strongest positive selection signal (ω = 136.369, *P* = 0.005), similar to owls, suggesting that falcons underwent a parallel adaptive evolution to dim-light conditions. While falcons are normally active in daytime, their hunting under relatively low-light conditions (e.g. crepuscularity) has been frequently reported^35^,^36^,^37^,^38^, supporting our molecular result. Adaptation to crepuscularity would result in selection for dim-light vision in falcons, and thus in addition to our molecular results we would expect their diurnal and crepuscular activities to also be reflected by their eye morphology (e.g. corneal diameters relative to axial lengths), which is demonstrated to be varied with different levels of ambient light in birds^12^,^13^. Moreover, by ancestral sequence reconstruction, we found falcons shared the two critical amino acid replacements (S167A in *LWS* and S223A in *SWS2*) found in owls that resulted in the short-wavelength shift of *LWS* and the long-wavelength shift of *SWS2* (Supplementary Figs 2 and 4). And particularly, parallel amino acid changes in both falcons and owls were found in another 15 sites of *SWS2* (Supplementary Fig. 4), in which 12 sites were under positive selection in the ancestral branch of owls (Supplementary Fig. 1), suggesting potential functional importance. The sharing of the same amino acid replacements between owls and falcons might imply a molecular convergence in these two opsin genes, possibly driven by adaption to similar crepuscular light conditions in both owls and falcons. In particular, the relatively strong molecular convergence in *SWS2* between falcons and owls observed may be due to similar adaption to the increased abundance of blue light during twilight^15^.

### Accipitriformes: Implication for high visual acuity and eye protection

Six genes (*CNGB3*, *CCDC66*, *CLN8*, *RPGR*, *NXNL2* and *COL2A1*) were found to be under positive selection among 120 vision genes examined in Accipitriformes. One of these genes, *CNGB3*, is involved in the phototransduction pathway in cones, while the other five genes are all involved in maintaining normal vision function. Specifically, using the branch-site test model, a strong positive selection of *CNGB3* (ω = 572.952, *P* = 0.046) along the ancestral branch of Accipitriformes was found. *CNGB3* encodes the *β* subunit of CNG channels (Fig. 2). The strong positive selection of *CNGB3* may enhance photoresponse and contribute to a high visual acuity. This is consistent with the members of Accipitriformes, e.g. eagles, possessing the highest visual acuity within Aves^6^,^39^. *CCDC66*, which demonstrated a relatively strong positive selection based on the branch-site model, is linked to the early development of the retina. A lack of *CCDC66* is found to cause early, slow progressive rod–cone dysplasia^40^. RPGR plays a critical role in cilia genesis, maintenance, and function, which is necessary for normal vision^41^. *CLN8* might play a role in transporting materials in and out of endoplasmic reticulum, and the *CLN8* mutation is linked to the progressive loss of vision^42^. *NXNL2* is a trophic factor for cone survival^43^. *COL2A1* is found to strengthen the vitreous body and support the retina^44^,^45^. The strong positive selection for maintaining the normal vision function in Accipitriformes may partly help them to cope with potential eye damage during hunting. Many members of Accipitriformes are known to catch ground-living animals, including large mammals^34^, and their eyes may incur injury when they crash into vegetation in pursuit of prey or suffer counterattack from prey^46^. Indeed, many cases of physical eye injuries in Accipitriformes have been reported in the field^29^,^30^, and this suggests that greater physical support of the eye is likely advantageous for survival.

### Coraciimorphae: Less modification in vision

**Figure 1:**
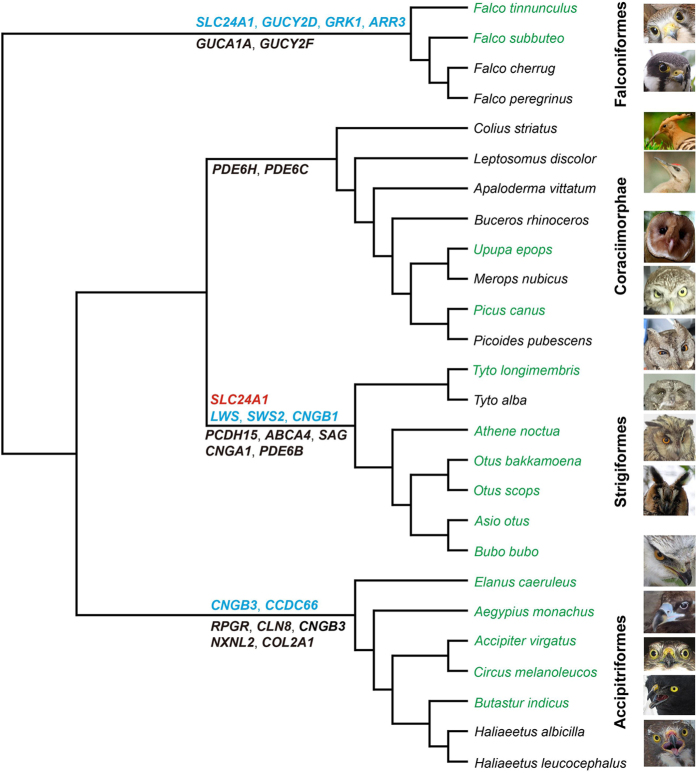
Positively selected genes identified in the four bird taxa (Strigifomres, Coraciimorphae, Falconiformes and Accipitriformes). The positively selected genes based on the branch model, branch-site model and the clade model are shown in red, blue and black, respectively. The species phylogenetic relationships are based on previous studies^1^,^49^,^50^,^51^. The 15 species under the transcriptome sequencing are highlighted in green, and their corresponding pictures are shown in the same order as that of their species names. The 11 species with vision gene data cited from GenBank are shown in black with no pictures.

**Figure 2:**
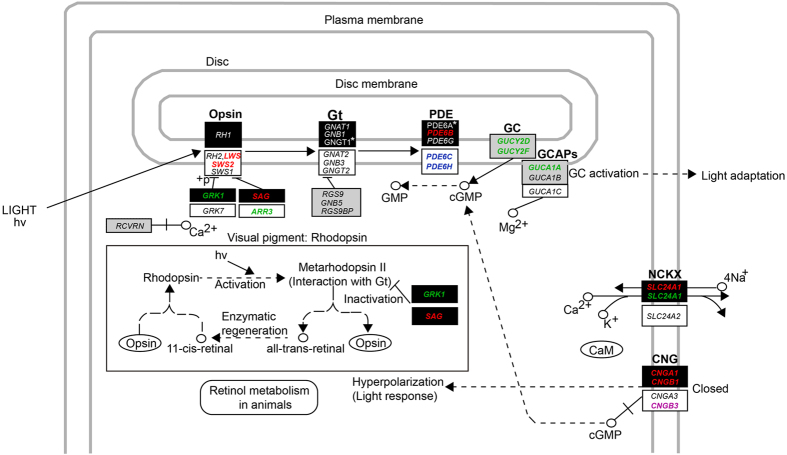
Positively selected genes involved in the phototransduction pathway in rods (according to KEGG pathway: map04744). For convenience, the genes involved in the phototransduction pathway in cones are also shown. Dark rectangles, white rectangles and grey rectangles show genes involved in the phototransduction pathway of rods, cones and both, respectively^7^. All the known principle genes involved in the phototransduction pathway are shown. The genes under investigation in this study are shown in italics. Positively selected genes found in Strigiformes, Falconiformes, Accipitriformes and Coraciimorphae are shown in red, green, violet and blue, respectively. *Shows two genes *GNGT1* and *PDE6A*, which have no transcripts found in our 15 species. The absence of *PDE6A* in birds found here is consistent with previous studies^56^,^57^,^58^. See text for the meanings of protein abbreviations and the functions of their corresponding genes. Solid line shows direct interaction and dashed line shows indirect interaction.

Coraciimorphae, including the bee-eaters, woodpeckers, hornbills, etc., is the closest extant relative of owls (Fig. 1), and its members are normally active by day. Among 114 genes examined in the eight species we studied in Coraciimorphae, only two genes, *PDE6C* and *PDE6H*, were found to be under positive selection based on the clade model, suggesting a divergent evolution of the two genes between Coraciimorphae and other taxa studied. The two genes play an essential role in cone phototransduction and respectively encode PDE hydrolytic subunit and inhibitory subunit (Fig. 2). The divergent evolution of the two PDE subunits may help to increase photoresponse through enhancing the activation and inactivation of PDE for a high visual acuity. Coraciimorphae is a highly diversified clade, and some, though not all, of its members inhabit woodland or forest where light level is relatively low, and an increased photoresponse would be, therefore, selectively favored.

## Conclusion

A comparative evolutionary analysis of 120 vision genes from four bird taxa was conducted based on retina transcriptome sequencing. Compared with their mainly diurnal relatives, more substantial visual modification was found in owls. A strong selection for nocturnal vision was revealed in owls, which may compensate for their losses of the genes involved in daylight or color vision, suggesting a sensory tradeoff. All PSGs found in falcons are involved in photoresponse recovery or light adaption, which is considered to be crucial for detection of motion. This is compatible with the behavioral styles of falcons as high-speed aerial hunters. Moreover, the PSGs for maintaining normal vision function were found in both owls and the distantly related Accipitriformes, which may be partly due to their high risk of eye injury during hunting. Additionally, one strongly positively selected gene, *CNGB3*, involved in the phototransduction cascade was detected in Accipitriformes, which may contribute to their high visual acuity. No PSGs were found in the ancestral branch of Coraciimorphae, the closest relative of owls, suggesting less modification in vision in this clade. Finally, one positively selected dim-light vision gene, *SLC24A1*, and two critical amino acid replacements leading to the short-wavelength shift of *LWS* and short-wavelength shift of *SWS2* were found to be shared between owls and falcons, implying their convergent or parallel evolution adapting to the similar crepuscular light condition.

## Methods

### Taxa covered

15 bird species were used for transcriptome sequencing in this study. The six owl species used in this study included both extant families (Tytonidae and Strigidae) of Strigiformes. One species (*Tyto longimembris*) was from Tytonidae and the other five species (*Athene noctua*, *Otus scops*, *Otus bakkamoena*, *Asio otus* and *Bubo bubo*) covered all three known subfamilies (Striginae, Surniinae and Asioninae) of Strigidae^47^. Two species (*Falco subbuteo* and *Falco tinnunculus*) of Falconiformes and five species from five genera of Accipitriformes including *Elanus caeruleus*, *Aegypius monachus*, *Accipiter virgatus, Circus melanoleucos* and *Butastur indicus* were used. We also included one species (*Upupa epops*) from Upupiformes and one species (*Picus canus*) from Piciformes, both belonging to the superorder Coraciimorphae, the most closely related relative to owls^1^. We also included 11 species closely related to our 15 study species (GenBank accession in Supplementary Table 4). These additional 11 species included the barn owl (*Tyto alba*), peregrine (*Falco peregrinus*), saker falcon (*Falco cherrug*), white-tailed eagle (*Haliaeetus albicilla*), bald eagle (*Haliaeetus leucocephalus*), speckled mousebird (*Colius striatus*), cuckoo roller (*Leptosomus discolor*), bar-tailed trogon (*Apaloderma vittatum*), rhinoceros hornbill (*Buceros rhinoceros*), carmine bee-eater (*Merops nubicus*) and downy woodpecker (*Picoides pubescens*).

### Retina tissue collection, RNA isolation and cDNA library construction

The retinal tissues used for transcriptome sequencing were obtained from one individual of each of the 15 species studied. The retinal tissues of each individual were rapidly excised and preserved in RNAlater and flash frozen in liquid nitrogen, and then stored at −80 °C until processed for RNA isolation. The 15 individuals used were injured individuals found in the field by our lab or the public from April to October, 2014. Before sacrificing, active individuals were transported to the laboratory and maintained under a natural light:dark condition at around 20 °C air temperature and 40% relative humidity, with food and water provided. These animals were sacrificed after between 12 to 24 hours and their retinal tissues were dissected during the daytime (10 am to 2 pm). All experimental procedures carried out in this study were approved by the National Animal Research Authority of Northeast Normal University, China (approval number: NENU-20080416) and the Forestry Bureau of Jilin Province of China (approval number: [2006]178). The experimental procedures were performed in accordance with an animal ethics approval granted by the Northeast Normal University.

Total RNA extractions were performed using TRIzol Reagent (Life Technologies), following the manufacturer’s protocol and instruction. 50–100 mg of tissue from each individual was used. The quality and quantity of RNA was assessed using Thermo Scientific NanoDrop 2000 (Thermo, Co) and Agilent 2100 Bioanalyzer (Agilent, Co). The retina cDNA sample was originally pooled with cochlear cDNA (used for hearing genes analyses) with an equal quality from the same individual for cDNA library construction. cDNA library from each individual was separately prepared using NEBNext Ultra RNA Library Prep Kit for Illumina (NEB, Co), according to manufacturer’s protocol. Specifically, Magnetic oligo (dT) beads (NEBNext Poly(A) mRNA Magnetic Isolation Module, NEB, Co) were used for enrichment of the poly (A) mRNA. The enriched mRNA was fragmented into small pieces, which were used for the synthesis of the first strand of cDNA by using superScript II reverse transcriptase and random hexamers, and subsequently the second strand of cDNA was synthesized by using buffer, dNTPs, RNaseH, and DNA polymerase I. The cDNA was purified using AMPure XP beads, and was subjected to end repair, adapter ligation and agarose gel electrophoresis filtration. Then, the suitable fragment size 250–350 bp was selected as templates for PCR amplification, which was used for cDNA library preparation. Paired-ending sequencing of cDNA library of all 15 species was performed using Illumina HiSeq2500 (Biomarker Technology, Co, Beijing) except for one species (*Otus bakkamoena*), in which its cDNA library was sequenced by Illumina HiSeq2000 (Beijing Institute of Genomics, CAS, Beijing).

### Data filtering and De Novo assembly

Raw reads were first preprocessed by removing adaptor sequences, and low-quality reads, this included reads with more than 10% unknown nucleotides and reads with the percentage of the bases with quality scores Q30 is lower than 15%, were removed. In this way, high-quality clean reads were obtained and used for *de novo* assembly. Reads from each library were assembled separately. De novo assembly was performed using Trinity software^10^ with default parameters except for min_kmer_cov >=2 was used, ensuring that only k-mers with at least 2x coverage was used. The longest transcript was used to yield unigenes and the unigenes longer than 200 bp were retained for further analyses. The transcriptome sequencing data have been deposited into the National Center for Biotechnology Information Sequence Read Archive database under accession numbers *Circus melanoleucos* (SRR3203217), *Asio otus*(SRR3203220), *Tyto longimembris* (SRR3203222), *Upupa epops*(SRR3203224), *Bubo bubo*(SRR3203225), *Elanus caeruleus* (SRR3203227), *Otus scops*(SRR3203230), *Falco tinnunculus*(SRR3203231), *Butastur indicus* (SRR3203233), *Accipiter virgatus*(SRR3203234), *Aegypius monachus*(SRR3203236), *Falco subbuteo*(SRR3203238), *Picus canus*(SRR3203240), *Athene noctua*(SRR3203242), *Otus bakkamoena*(SRR3203243).

### Vision gene sequences and alignment

*Gallus gallus* annotated vision genes were retrieved from Aimgo (http://amigo.geneontology.org/amigo) using “vision” as a search keyword. Using this approach, 124 vision genes were found. Subsequently, the protein sequences of the 124 genes of *Gallus gallus* were used as the query sequences to blast against each of our 15 species unigene pools using Tblastn 2.2.29 with an e-value cut-off of 1e^−5^. The unigene sequences obtained were then annotated by blasting against the NCBI nr/nt database using the online Blastn, and only the unigene sequences with the same gene annotation as that of the query sequences of *Gallus gallus* were used for subsequent analyses. In addition to the vision gene list from Aimgo, an additional 18 vision genes involved in the phototransduction cascade based on previous literature^7^ were included. To extract the 18 gene sequences from each of our 15 species unigene pools, we first downloaded the sequences of the corresponding genes from relatives (e.g. *Falco peregrinus, Falco cherrug* and *Gallus gallus*) of our 15 species, and subsequently these sequences were used as the query sequences to blast against our 15 species unigene pools by using Blastn 2.2.29. Using the two approaches, we identified 142 vision genes in total. In addition to the 142 vision genes from our 15 species, we also downloaded the sequences of another 11 species mentioned above from NCBI using the published accession numbers.

Vision gene sequences were aligned using ClustalW algorithm that is implemented in the DNAman software (version 8.0, Lynnon BioSoft), and was manually inspected one by one for quality. Species with short sequences or low sequence identities (e.g., long indels and multiple ambiguous bases Ns) were removed. In addition, the genes shared by no more than two of our four focal bird taxa were excluded for subsequent analyses. After such pruning, a total of 120 vision genes with high-quality alignment results were retained and their coding sequences were used for subsequent positive selection analyses.

### Positive selection analyses

Adaptive evolution of vision genes along the ancestral branches and clades of Strigiformes, Coraciimorphae, Falconiformes and Accipitriformes were analyzed using a maximum-likelihood method implemented in the Codeml program in PAML 4.8a package^48^. For the adaptive evolution analyses, an unrooted species tree ((Strigiformes, Coraciimorphae), Falconiformes, Accipitriformes) was constructed based on previous studies^1^,^2^ and the species relationships within each of the four taxa followed previous studies^49^,^50^,^51^. For the analyses, the branch model, branch-site model and clade model C were employed with each of the four focal taxa respectively treated as the foreground branch or foreground clade while all others used as the background branches or clades. Likelihood ratio tests (LRT) were used to test whether the more complex model fits better than the simpler, nested models. Specifically, for the branch model, to test whether the foreground branches of interest have a different ratio from the background branches, the two-ratio branch model and the one-ratio branch model were used. To further test whether the foreground branches have a ω that is greater than one, the likelihood values of the two-ratio branch model with and without the constraint ω = 1 were compared.

In addition to the branch model, we also used the branch-site model to detect positively selected sites along the foreground branches that were pre-specified. For this, Test 2, which compares the modified model A with the null model, with ω = 1 fixed, was used. In the modified model A, four classes of sites are assumed and only the foreground branches are assumed to have experienced positive selection. Among the four classes of sites, site class 0 (0 < ω_0_ < 1) includes codons that are conserved throughout the tree, and site class 1 (ω_1_ = 1) includes evolutionarily neutral codons. Site classes 2a and 2b include codons that are evolutionarily conserved (0 < ω_0_ < 1) or neutral (ω_1_ = 1) on the background branches, respectively, but are assumed to be under positive selection (ω_2_ > 1) on the foreground branches. When the LRT suggests positive selection, the Bayes empirical Bayes method was used to estimate the posterior probabilities that each codon belongs to the site class of positive selection on the foreground branches.

Clade model C was used to identify the codons under divergent selection pressure between the foreground clade and the background clade. The model assumes variation in ω among sites, allowing a fraction of sites evolving under divergent selective pressures. In the model, three classes of sites are assumed, and the first two classes of sites respectively represent evolutionary conserved or neural codons, while the third site class represents codons under divergent selection pressures between foreground clade and the background clade. Model M1a was used as the null model to test for the existence of divergently selected sites.

### Robustness tests of positive selection genes

After positively selected genes (PSGs) were identified based on the branch model, branch-site model and clade model C mentioned above, these PSGs were further analyzed for robustness as several factors have been known to affect the result of positive selection analyses and lead to false positive results. **I**) To guarantee that only the orthologous gene sequences were used, the annotations of PSGs sequences from all species studied were confirmed by blasting their cDNA sequences and amino acid sequences against NCBI nr/nt database using the online Blastn and Blastp with the default parameter settings. **II**) For the PSGs identified based on the clade model C, we further used a revised null model (M2a_rel) for LRT since M2a_rel is considered to better account for among-site variation in selective constraint and to reduce false positives compared with the standard null model M1a^52^. **III**) We realigned the gene sequences of PSGs using webPRANK (http://www.ebi.ac.uk/goldman-srv/webprank/)^53^ and made a reanalysis of positive selection based on the certain models under which PSGs were identified. PRANK is suggested to generate a more reliable alignment than other alignment methods and have the lowest false-positive rates during the branch-site inference of selection^54^. **IV**) Recombination is considered to lead to a high rate of false positive in positive selection inference^55^. We screened for the evidence of recombination in PSGs (Supplementary Table 6) using GARD program implemented in Datamonkey webserver (http://www.datamonkey.org/) and reanalyzed their positive selection based on the gene partitions of those PSGs in which the recombination events were found. **V**) To avoid being trapped at a local optimum, we respectively changed initial parameter values of kappa and omega to see whether the results of the positive selection inference were stable. After examining of PSGs by the five steps, any PSGs that did not pass any one of the five tests were excluded for subsequent analyses, and in this way about half of original PSGs were filtered out and 22 PSGs retained.

### Ancestral sequence reconstruction and spectral tuning analyses

Ancestral amino acid sequences of four visual opsins (*LWS*, *SWS2*, *RH2* and *RH1*) were reconstructed at all ancestral nodes to analyze the spectral changes along different branches. Ancestral sequences reconstructions were carried out using the empirical Bayes approach implemented in PAML 4.8^17^, using amino acid-based marginal reconstruction. In the marginal reconstruction, the character was assigned to one single interior node and the character with the highest posterior probability is considered to be the best reconstruction. For the reconstruction, the amino acid substitution model of JTT and Poisson model were employed. The empirical model JTT assumes different amino acids have different substitution rates while the Poisson model assumes every amino acid has the same rate of changing into any other amino acid. The results based on the JTT and Poisson models were highly similar, and for convenience only the results based on JTT model are shown. As the ancestral sequences were achieved for each internal node, the corresponding amino acid changes along different branches were identified. Since amino acid sites numbering linked to spectral tuning are normally based on the bovine rhodopsin sequence, we aligned our sequences against the bovine rhodopsin sequence (NP_001014890) to check the location of amino acid replacement in those critical amino acid sites. After the amino acid replacements in those critical amino acid sites along different branches were identified, the effects of the amino acid replacement on spectral tuning were examined following previous studies (Supplementary Table 5).

### Cloning and sequencing of the *RH1* gene

To assess the quality of our assembled unigenes, the *RH1* gene was selected to be sequenced by cloning. For this, the liver tissues from the same 15 individuals of the 15 species used for transcriptome sequencing were used. Genomic DNA was extracted from ethanol-preserved liver tissue using the Ezup Column Animal Genomic DNA Purification Kit (Sangon, Shanghai). Three primer pairs were used to amplify the complete coding sequence of *RH1* gene, (Supplementary Table 7). One primer pair for amplification of *RH1* exons 2–4 was used from the literature and two primer pairs for amplification of *RH1* exons 1, 4–5 were designed by this study using Primer Premier 5.0. To reduce mutations introduced by amplification, Q5TM super fidelity 2× Master Mix (Biolabs) was used following manufacturer’s instruction. The different exons of RH1 gene were respectively amplified in a total volume of 25 μl using l μl of template DNA, 1.25 μl of each primer (10 μM), 12.5 μl Q5^TM^ super fidelity 2× Master Mix and 9 μl ddH_2_O. PCR reactions consisted of an initial denaturation at 98 °C for 30 s, followed by 35 cycles of 98 °C for 10 s, 54–63.3 °C for 30 s (Supplementary Table 7), 72 °C for about 1 min and a final 2 min extension at 72 °C. PCR products were purified using MiniBEST Agarose Gel DNA Extraction Kit (TaKaRa, Dalian). The purified PCR products were used for cloning sequencing by using pESY-Blunt Zero Cloning Kit (TransGen, Beijing). Five positive clones of each individual were randomly selected to be sequenced by an ABI 3730 XL automated sequencer (Applied Biosystems) (Sangon, Shanghai).

Partial or complete coding sequences of the *RH1* gene were obtained from the 15 individuals used in the transcriptome sequencing (Supplementary Table 2). Subsequently, the sequence of each individual was aligned with the corresponding transcript sequence from the same individual and their sequence identities were respectively calculated using DNAman software. The sequence identity ranged from 99.23% to 99.92%, with an average of 99.60% (Supplementary Table 2).

## Additional Information

**How to cite this article**: Wu, Y. *et al.* Retinal transcriptome sequencing sheds light on the adaptation to nocturnal and diurnal lifestyles in raptors. *Sci. Rep.* **6**, 33578; doi: 10.1038/srep33578 (2016).

## Supplementary Material

Supplementary Information

Supplementary Dataset 1

## Figures and Tables

**Table 1:** **Positively selected genes identified in the four bird taxa.**

Taxa /Genes	Parameter estimates	2∆L	df	*p-value*
Strigiformes				
*SLC24A1*	***ω***_***1***_** = 999**	9.17	1	0.002
*LWS*	*p*_*0*_ = 0.928 *p*_*1*_ = 0.021 *p*_*2a*_ = 0.050 *p*_*2b*_ = 0.001	17.66	1	2.647E-05
	*ω*_*0*_ = 0.032 *ω*_*1*_ = 1.000 ***ω***_***2a***_ = **51.224** ***ω***_***2b***_** = 51.224**			
*SWS2*	*p*_*0*_ = 0.785 *p*_*1*_ = 0.094 *p*_*2a*_ = 0.108 *p*_*2b*_ = 0.013	16.69	1	4.391E-05
	*ω*_*0*_ = 0.041 *ω*_*1*_ = 1.000 ***ω***_***2a***_ = **29.447** ***ω***_***2b***_** = 29.447**			
*CNGB1*	*p*_*0*_ = 0.798 *p*_*1*_ = 0.142 *p*_*2a*_ = 0.051 *p*_*2b*_ = 0.009	6.10	1	0.014
	*ω*_*0*_ = 0.042 *ω*_*1*_ = 1.000 ***ω***_***2a***_ = **5.081** ***ω***_***2b***_** = 5.081**			
*PCDH15*	*p*_*0*_ = 0.819 *p*_*1*_ = 0.103 *p*_*2*_ = 0.079	42.87	1	5.853E-11
	*ω*_*0*_ = 0.032 *ω*_*1*_ = 1.000 ***ω***_***2***_** = 2.121**			
*ABCA4*	*p*_*0*_ = 0.784 *p*_*1*_ = 0.102 *p*_*2*_ = 0.115	23.57	1	1.206E-06
	*ω*_*0*_ = 0.063 *ω*_*1*_ = 1.000 ***ω***_***2***_** = 2.025**			
*SAG*	*p*_*0*_ = 0.664 *p*_*1*_ = 0.162 *p*_*2*_ = 0.175	16.54	1	4.759E-05
	*ω*_*0*_ = 0.007 *ω*_*1*_ = 1.000 ***ω***_***2***_** = 1.933**			
*CNGA1*	*p*_*0*_ = 0.879 *p*_*1*_ = 0.058 *p*_*2*_ = 0.062	25.25	1	5.042E-07
	*ω*_*0*_ = 0.017 *ω*_*1*_ = 1.000 ***ω***_***2***_** = 1.720**			
*PDE6B*	*p*_*0*_ = 0.891 *p*_*1*_ = 0.000 *p*_*2*_ = 0.109	6.96	1	0.008
	*ω*_*0*_ = 0.015 *ω*_*1*_ = 1.000 ***ω***_***2***_** = 1.374**			
Falconiformes				
*SLC24A1*	*p*_*0*_ = 0.679 *p*_*1*_ = 0.314 *p*_*2a*_ = 0.005 *p*_*2b*_ = 0.002	7.89	1	0.005
	*ω*_*0*_ = 0.068 *ω*_*1*_ = 1.000 ***ω***_***2a***_** = 136.369** ***ω***_***2b***_** = 136.369**			
*GUCY2D*	*p*_*0*_ = 0.930 *p*_*1*_ = 0.063 *p*_*2a*_ = 0.007 *p*_*2b*_ = 0.000	4.33	1	0.037
	*ω*_*0*_ = 0.029 *ω*_*1*_ = 1.000 ***ω***_***2a***_ = **65.241** ***ω***_***2b***_** = 65.241**			
*GRK1*	*p*_*0*_ = 0.876 *p*_*1*_ = 0.099 *p*_*2a*_ = 0.023 *p*_*2b*_ = 0.003	9.03	1	0.003
	*ω*_*0*_ = 0.029 *ω*_*1*_ = 1.000 ***ω***_***2a***_ = **36.362** ***ω***_***2b***_** = 36.362**			
*ARR3*	*p*_*0*_ = 0.937 *p*_*1*_ = 0.049 *p*_*2a*_ = 0.013 *p*_*2b*_ = 0.001	4.87	1	0.027
	*ω*_*0*_ = 0.041 *ω*_*1*_ = 1.000 ***ω***_***2a***_ = **21.894** ***ω***_***2b***_** = 21.894**			
*GUCA1A*	*p*_*0*_ = 0.913 *p*_*1*_ = 0.000 *p*_*2*_ = 0.087	9.86	1	0.002
	*ω*_*0*_ = 0.008 *ω*_*1*_ = 1.000 ***ω***_***2***_** = 2.057**			
*GUCY2F*	*p*_*0*_ = 0.810 *p*_*1*_ = 0.000 *p*_*2*_ = 0.189	11.21	1	0.001
	*ω*_*0*_ = 0.075 *ω*_*1*_ = 1.000 ***ω***_***2***_** = 1.903**			
Accipitriformes				
*CNGB3*	*p*_*0*_=0.818 *p*_*1*_ = 0.180 *p*_*2a*_ = 0.002 *p*_*2b*_ = 0.000	3.97	1	0.046
	*ω*_*0*_ = 0.035 *ω*_*1*_ = 1.000 ***ω***_***2a***_** = 572.952** ***ω***_***2b***_** = 572.952**			
*CCDC66*	*p*_*0*_ = 0.649 *p*_*1*_ = 0.305 *p*_*2a*_ = 0.032 *p*_*2b*_ = 0.015	5.08	1	0.024
	*ω*_*0*_ = 0.275 *ω*_*1*_ = 1.000 ***ω***_***2a***_ = **44.842** ***ω***_***2b***_** = 44.842**			
*RPGR*	*p*_*0*_ = 0.797 *p*_*1*_ = 0.193 *p*_*2*_ = 0.011	6.11	1	0.013
	*ω*_*0*_ = 0.064 *ω*_*1*_ = 1.000 ***ω***_***2***_** = 17.029**			
*CLN8*	*p*_*0*_ = 0.816 *p*_*1*_ = 0.156 *p*_*2*_ = 0.028	6.32	1	0.012
	*ω*_*0*_ = 0.035 *ω*_*1*_ = 1.000 ***ω***_***2***_** = 9.626**			
*CNGB3*	*p*_*0*_ = 0.881 *p*_*1*_ = 0.023 *p*_*2*_ = = 0.097	9.57	1	0.002
	*ω*_*0*_ = 0.064 *ω*_*1*_ = 1.000 ***ω***_***2***_** = 4.375**			
*NXNL2*	*p*_*0*_ = 0.760 *p*_*1*_ = 0.000 *p*_*2*_ = 0.240	11.17	1	0.001
	*ω*_*0*_ = 0.012 *ω*_*1*_ = 1.000 ***ω***_***2***_** = 2.823**			
*COL2A1*	*p*_*0*_ = 0.891 *p*_*1*_ = 0.000 *p*_*2*_ = 0.109	82.40	1	1.110E-19
	*ω*_*0*_ = 0.000 *ω*_*1*_ = 1.000 ***ω***_***2***_** = 1.589**			
Coraciimorphae				
*PDE6H*	*p*_*0*_ = 0.843 *p*_*1*_ = 0.077 *p*_*2*_ = 0.080	4.86	1	0.028
	*ω*_*0*_ = 0.054 *ω*_*1*_ = 1.000 ***ω***_***2***_** = 5.878**			
*PDE6C*	*p*_*0*_ = 0.910 *p*_*1*_ = 0.074 *p*_*2*_ = 0.016	10.24	1	0.001
	*ω*_*0*_ = 0.035 *ω*_*1*_ = 1.000 ***ω***_***2***_** = 4.182**			

With the positive selection analyses, each of the four taxa was respectively treated as the foreground branch or clade while the other three were used as the background one. For convenience, only the ω values for the foreground branches or clades are shown. All the positively selected genes based on the branch model, branch-site model and clade model C are listed (please see Fig. 1 for the genes classes). Note that for the likelihood ratio tests under the clade model C, the M2a_real was used as the null model.

2∆L: twice difference of likelihood values between two nested models; df: degrees of freedom; *p*: proportion of sites in different site classes. The four site classes (*p*_*0*_, *p*_*1*_, *p*_*2a*_ and *p*_*2b*_) of the branch-site model and the three site classes (*p*_*0*_, *p*_*1*_ and *p*_*2*_) of the clade model C are shown.
