# Corrigendum: Retinal transcriptome sequencing sheds light on the adaptation to nocturnal and diurnal lifestyles in raptors

**DOI:** 10.1038/srep35895

**Published:** 2016-11-08

**Authors:** Yonghua Wu, Elizabeth A. Hadly, Wenjia Teng, Yuyang Hao, Wei Liang, Yu Liu, Haitao Wang

Scientific Reports 6: Article number: 33578; 10.1038/srep33578 published online: 09 20 2016; updated: 11 08 2016.

This Article contains a typographical error in the Conclusion section,

“Finally, one positively selected dim-light vision gene, *SLC24A1*, and two critical amino acid replacements leading to the short-wavelength shift of *LWS* and short-wavelength shift of *SWS2* were found to be shared between owls and falcons, implying their convergent or parallel evolution adapting to the similar crepuscular light condition”.

should read:

“Finally, one positively selected dim-light vision gene, *SLC24A1*, and two critical amino acid replacements leading to the short-wavelength shift of *LWS* and long-wavelength shift of *SWS2* were found to be shared between owls and falcons, implying their convergent or parallel evolution adapting to the similar crepuscular light condition”.

