# Supplementary material for: Retinal transcriptome sequencing sheds light on the adaptation to nocturnal and diurnal lifestyles in raptors
Source: Sci Rep. 2016 Sep 20;6:33578. doi: 10.1038/srep33578 (PMC5028738; doi:10.1038/srep33578)
Supplement: Supplementary Information [file srep33578-s1.pdf]

# Supplementary Information

## Retinal transcriptome sequencing sheds light on the adaptation to nocturnal and diurnal lifestyles in raptors

Yonghua Wu, Elizabeth A. Hadly, Wenjia Teng, Yuyang Hao, Wei Liang, Yu Liu, Haitao Wang

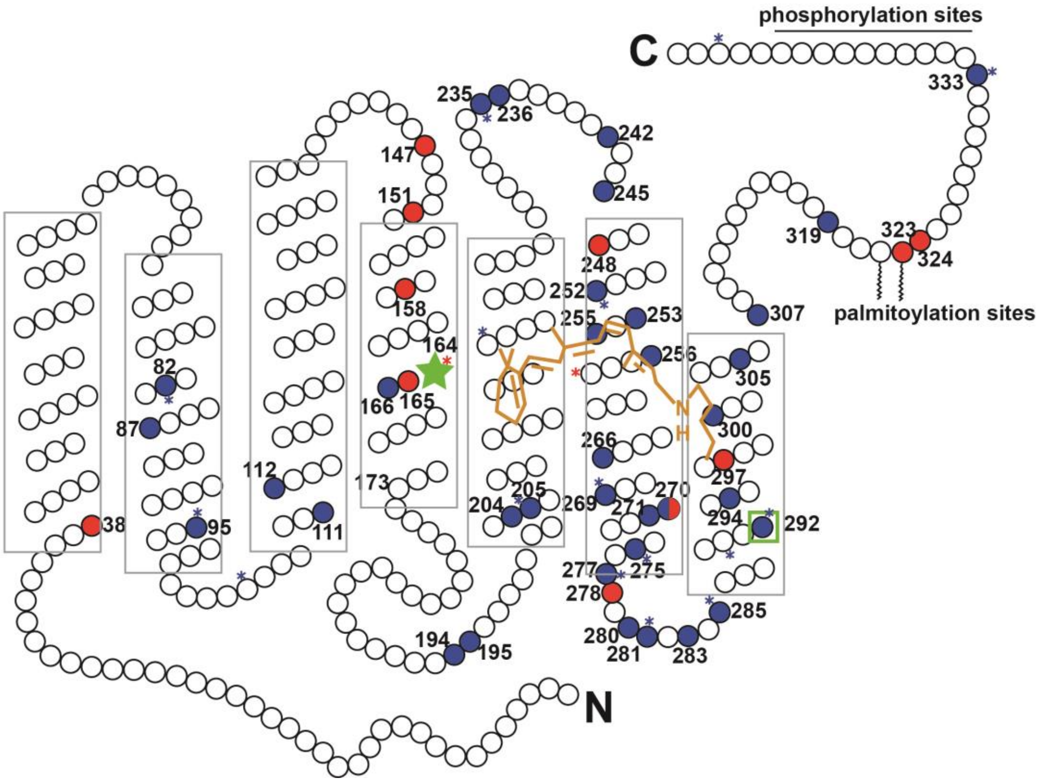


**Supplementary Figure 1 The positively selected sites of *LWS* (red) and *SWS2* (blue) mapping on the secondary structure of bovine rhodopsin.** The numbering is based on the bovine rhodopsin. The model is based on previous studies[16,59-60]. The spectral tuning sites of *LWS* and *SWS2* found in this study are shown in a green star and a green square, respectively. The 11-cis-retinal is shown in orange. Red * and blue * show amino acid sites of *LWS* and *SWS2* under parallel amino acid changes between owls and falcons, respectively. The grey rectangle shows the even transmembrane domains of rhodopsin.

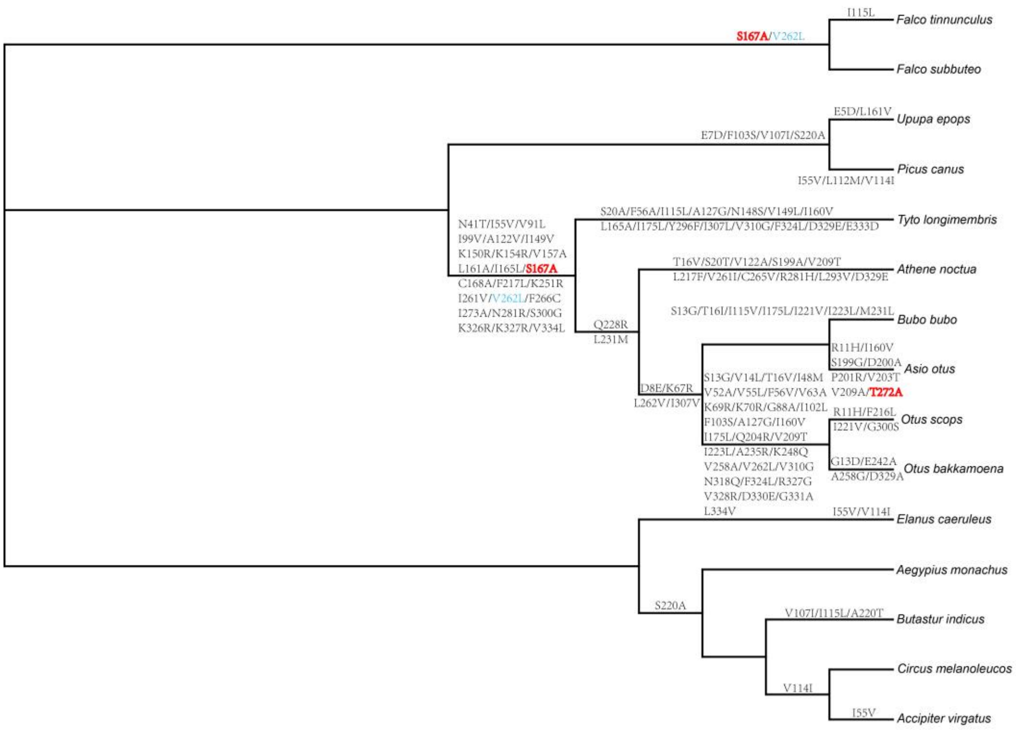


**Supplementary Figure 2 Amino acid replacements of *LWS* along different branches.** Phylogenetic relationships among species followed previous studies[1,49-51]. The critical amino acid replacements that are considered to be responsible for the spectral tuning are highlighted in red. Blue shows parallel amino acid changes between ancestral branches of owls and falcons.

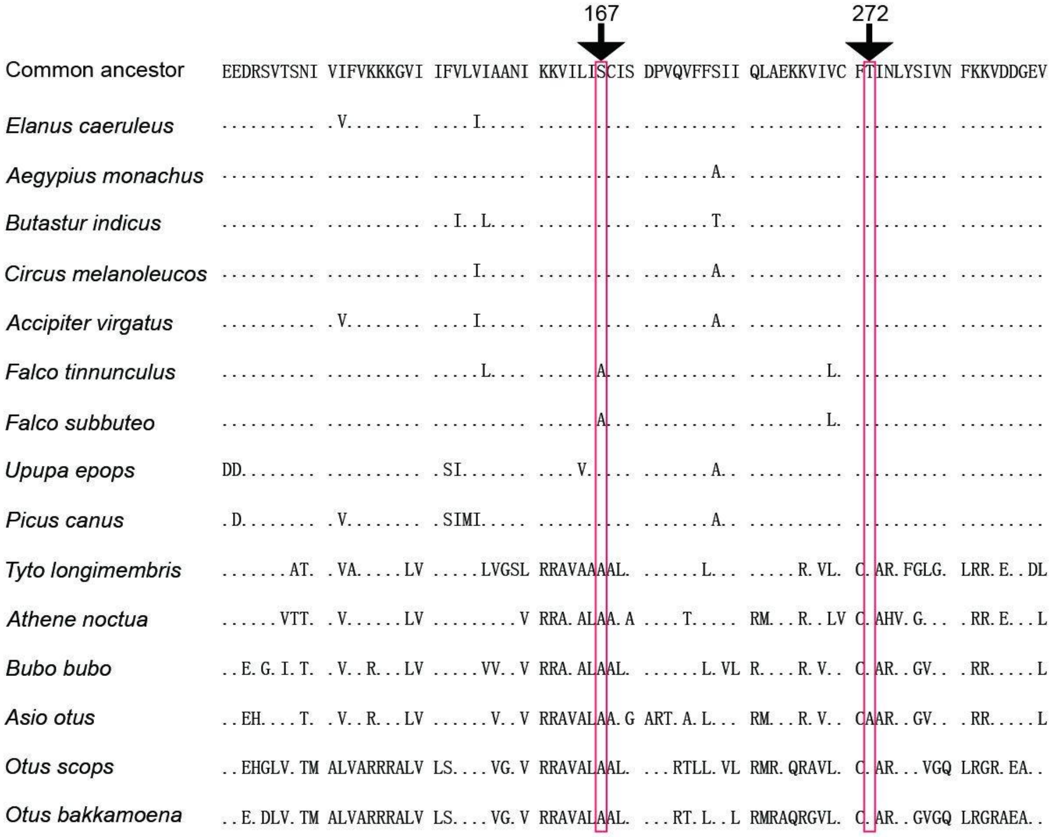


**Supplementary Figure 3 Variable amino acid sites of *LWS* in 15 species and their common ancestor.** The dot shows identical amino acid to the common ancestor. Two amino acid sites associated with the *LWS* spectral turning are highlighted.

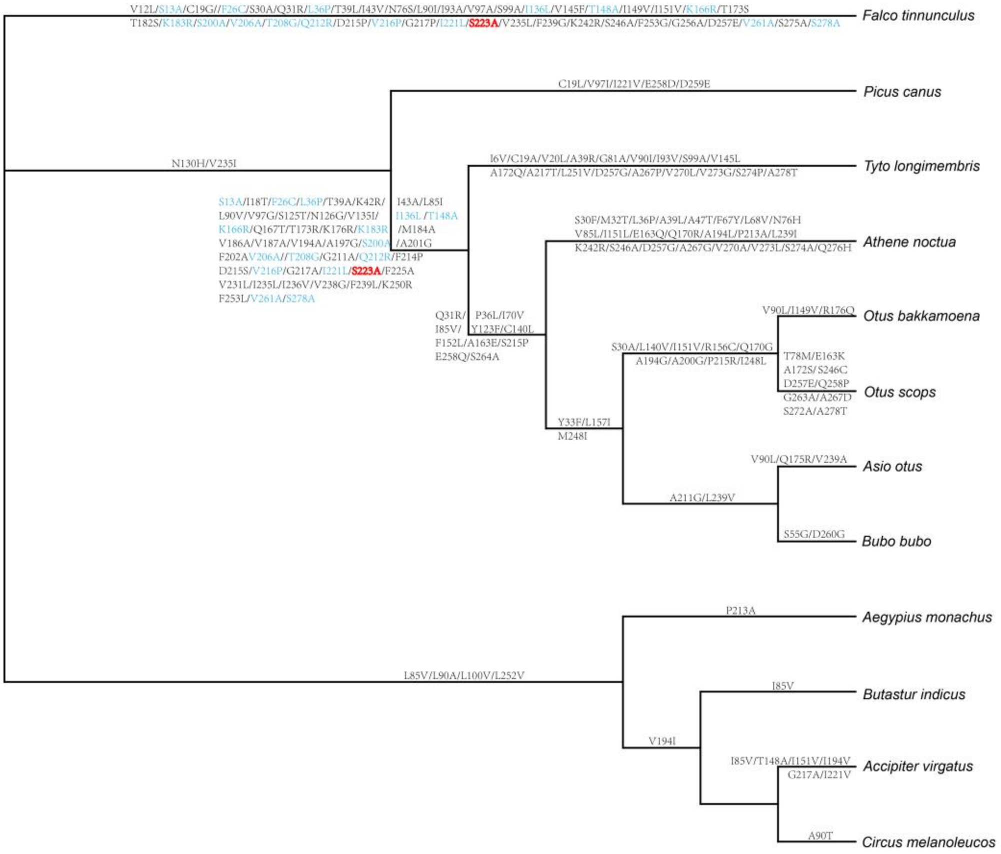


**Supplementary Figure 4 Amino acid replacements of *SWS2* along different branches.** Phylogenetic relationships among species followed previous studies[1,49-51]. The amino acid replacements associated with the *SWS2* spectral tuning are highlighted in red. Blue shows parallel amino acid changes between ancestral branches of owls and falcon branch.

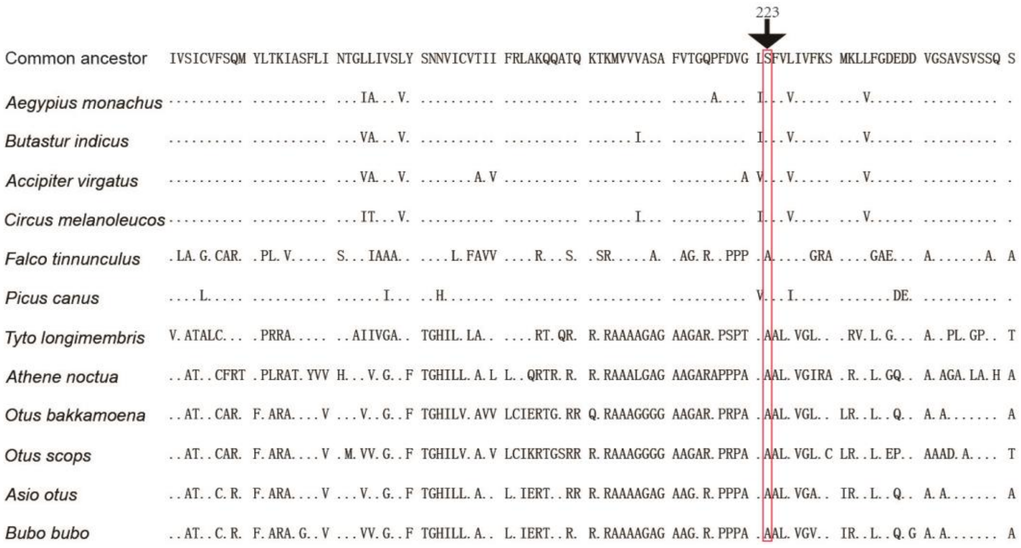


**Supplementary Figure 5 Variable amino acid sites of SWS2 in 12 species and their common ancestor.** The dot shows identical amino acid to the common ancestor. The amino acid site associated with the *SWS2* spectral turning is highlighted.

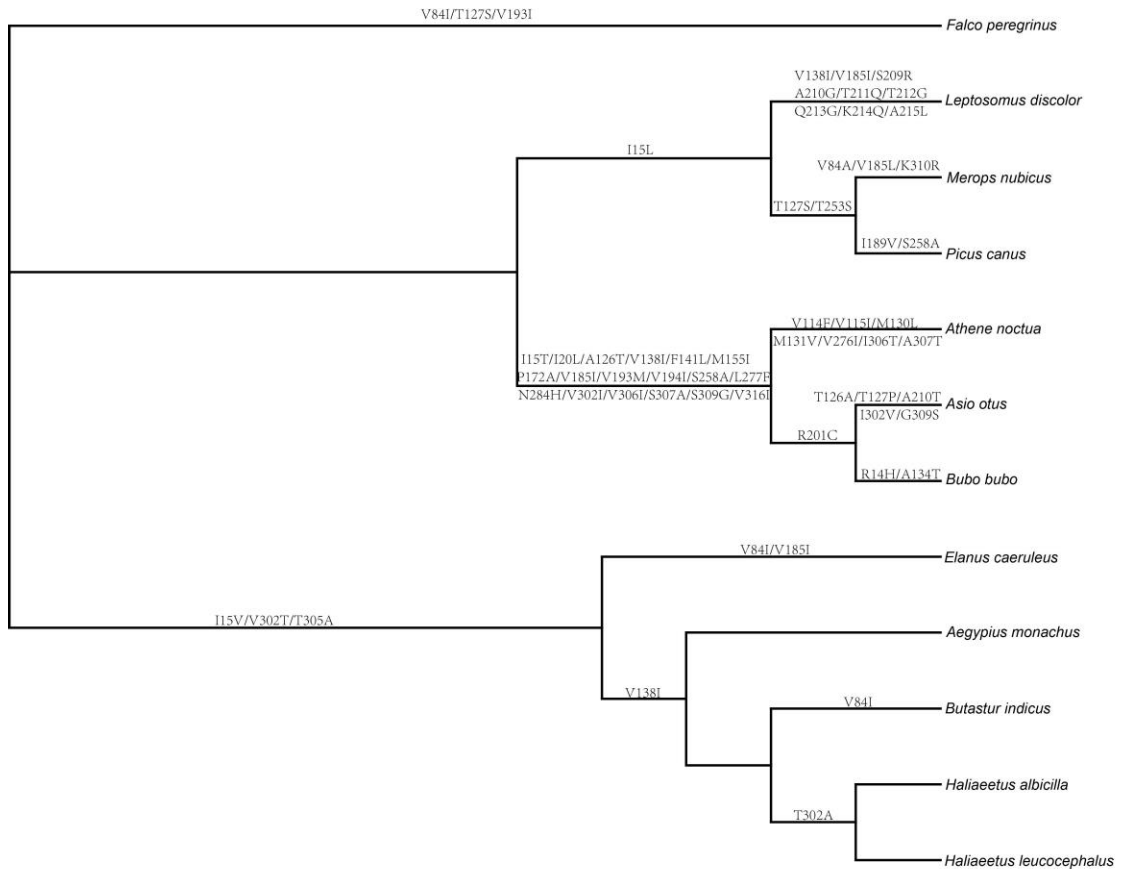


**Supplementary Figure 6 Amino acid replacements of *RH2* along different branches.** Phylogenetic relationships among species followed previous studies[1,49-51]. No amino acid replacement associated with the *RH2* spectral turning is found.

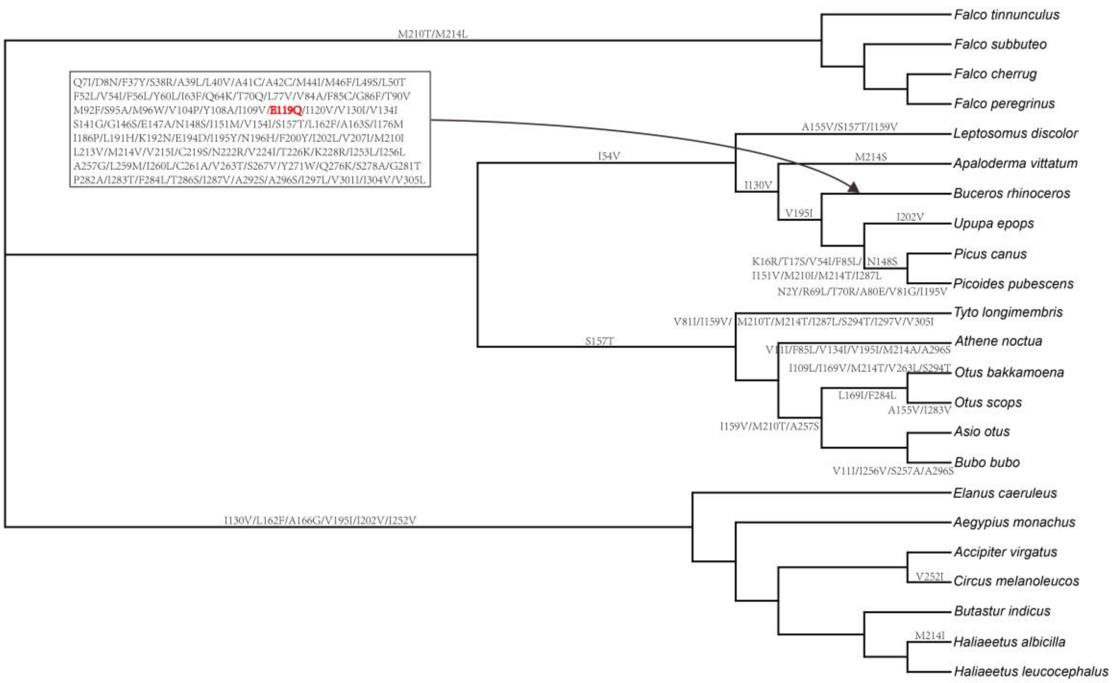


**Supplementary Figure 7 Amino acid replacements of *RH1* along different branches.** Phylogenetic relationships among species followed previous studies[1,49-51]. The amino acid replacement associated with the *RH1* spectral tuning is highlighted in red. The amino acid replacement E119Q (corresponding to E122Q in bovine rhodopsin) along the branch of rhinoceros hornbill (*Buceros rhinoceros*) is considered to reduce λmax of *RH1* by 20 nm (Supplementary Table 5).

**Supplementary Table 1 The basic statistics of the transcriptome sequencing for 15 species studied.**

| Species/Average | Raw reads (No.) | Clean reads (No.) | Clean bases (bp) | Unigene (No.) | Mean length (bp) | N50 (bp) |
|---|---|---|---|---|---|---|
| *Falco tinnunculus* | 51627942 | 50327827 | 12670306819 | 181077 | 681 | 1271 |
| *Falco subbuteo* | 51265913 | 50094480 | 12608048272 | 196648 | 679 | 1208 |
| *Upupa epops* | 46289873 | 45071285 | 11340043000 | 128819 | 695 | 1437 |
| *Picus canus* | 59253971 | 58407841 | 14707622487 | 160139 | 640 | 1082 |
| *Tyto longimembris* | 47123024 | 44590406 | 11209515376 | 169590 | 620 | 992 |
| *Athene noctua* | 54430631 | 53735717 | 13532670717 | 162550 | 693 | 1333 |
| *Otus bakkamoena* | 67375596 | 67324756 | 6715503299 | 190941 | 714 | 1359 |
| *Otus scops* | 50255927 | 47780582 | 12020208310 | 162940 | 666 | 1216 |
| *Asio otus* | 44516155 | 43716816 | 11006115275 | 161612 | 693 | 1325 |
| *Bubo bubo* | 51964845 | 50656163 | 12747030463 | 160561 | 674 | 1255 |
| *Elanus caeruleus* | 53858822 | 52591133 | 13235187097 | 191698 | 673 | 1212 |
| *Aegypius monachus* | 49449600 | 47841645 | 12036588777 | 178557 | 653 | 1155 |
| *Accipiter virgatus* | 48522401 | 46686932 | 11743460419 | 168270 | 662 | 1204 |
| *Circus melanoleucos* | 49208081 | 48228181 | 12141863212 | 182491 | 649 | 1152 |
| *Butastur indicus* | 49568683 | 48503775 | 12210776879 | 165290 | 706 | 1423 |
| Average | 51647431 | 50370503 | 11994996027 | 170746 | 673 | 1242 |

**Supplementary Table 2 Identity of coding sequence of *RH1* gene based on the transcriptome sequencing and cloning sequencing.** The fragment lengths of each species obtained by cloning sequencing are shown.

| **Species** | **Length (bp)** | **Identity (%)** |
|---|---|---|
| *Falco tinnunculus* | 880 | 99.55 |
| *Falco subbuteo* | 713 | 99.72 |
| *Upupa epops* | 333 | 99.40 |
| *Picus canus* | 519 | 99.42 |
| *Tyto longimembris* | 519 | 99.23 |
| *Athene noctua* | 519 | 99.42 |
| *Otus bakkamoena* | 1053 | 99.92 |
| *Otus scops* | 1053 | 99.43 |
| *Asio otus* | 1053 | 99.72 |
| *Bubo bubo* | 1053 | 99.72 |
| *Elanus caeruleus* | 696 | 99.71 |
| *Aegypius monachus* | 880 | 99.66 |
| *Accipiter virgatus* | 882 | 99.55 |
| *Circus melanoleucos* | 1053 | 99.81 |
| *Butastur indicus* | 882 | 99.77 |

**Supplementary Table 3 The 120 vision genes examined for the four bird taxa in this study.** Dots show the available gene sequences and red crosses show the unavailable gene sequences.

| Gene symbols | Strigiformes | Accipitriformes | Falconiformes | Coraciimorphae |
|---|---|---|---|---|
| *ABCA4* | • | • | • | • |
| *ARR3* | • | • | • | • |
| *ATP8A2* | • | • | • | • |
| *ATP8B1* | • | • | • | • |
| *BBS4* | • | • | • | • |
| *BHLHE23* | • | • | • | • |
| *CACNA2D4* | • | • | • | • |
| *CACNB2* | • | • | • | • |
| *CACNB4* | • | • | • | • |
| *CCDC66* | • | • | • | • |
| *CHRNB2* | • | • | • | • |
| *CLN5* | • | • | • | • |
| *CLN6* | • | • | • | • |
| *CLN8* | • | • | • | • |
| *CNGA1* | • | • | • | • |
| *CNGA3* | • | • | • | • |
| *CNGB1* | • | • | • | • |
| *CNGB3* | • | • | • | • |
| *CNIH2* | • | • | • | • |
| *COL11A1* | • | • | X | • |
| *COL2A1* | • | • | • | X |
| *CRABP1* | • | • | • | • |
| *CRABP2* | • | • | • | • |
| *CRDS2* | • | • | • | • |
| *CRYBA1* | • | • | • | • |
| *CRYBB2* | • | • | • | • |
| *DMD* | • | • | • | • |
| *DNAJC19* | • | • | • | • |
| *EPAS1* | • | • | • | • |
| *EPHB2* | • | • | • | X |
| *EYS* | • | • | • | X |

| | | | | |
|---|---|---|---|---|
| *GABRR2* | • | • | • | • |
| *GJA10* | • | • | • | • |
| *GJC1* | • | • | • | • |
| *GJD2* | • | • | • | • |
| *GLRA1* | • | • | • | • |
| *GLRB* | • | • | • | • |
| *GNAT1* | • | • | • | • |
| *GNAT2* | • | • | • | • |
| *GNB1* | • | • | • | • |
| *GNB3* | • | • | • | • |
| *GNB5* | • | • | • | • |
| *GNGT2* | • | • | • | • |
| *GPR98* | • | • | • | • |
| *GRK1* | • | • | • | X |
| *GRK7* | • | • | • | • |
| *GUCA1A* | • | • | • | • |
| *GUCA1B* | • | • | • | • |
| *GUCA1C* | • | • | • | • |
| *GUCY2D* | • | • | • | • |
| *GUCY2F* | • | • | • | • |
| *HBEGF* | • | • | • | • |
| *HCN1* | • | • | X | • |
| *ISL1* | • | • | • | X |
| *KCNA2* | • | • | • | • |
| *LAMB2* | • | • | • | • |
| *LAMC3* | • | • | • | • |
| *LUM* | • | • | • | • |
| *LWS* | • | • | • | • |
| *MYO5A* | • | • | • | • |
| *MYO7A* | • | • | • | • |
| *NAV2* | • | • | • | • |
| *NOB1* | • | • | • | • |
| *NR2E1* | • | • | • | • |
| *NRP1* | • | • | • | • |

| | | | | |
|---|---|---|---|---|
| *NRP2* | • | • | • | • |
| *NTRK2* | • | • | • | • |
| *NXNL2* | • | • | • | • |
| *NYX* | • | • | • | • |
| *OPA1* | • | • | • | • |
| *OPN3* | • | • | • | • |
| *OPN4-1* | • | • | • | • |
| *OPN5* | • | • | • | • |
| *PCDH15* | • | • | • | • |
| *PDCL* | • | • | • | • |
| *PDE6B* | • | • | • | • |
| *PDE6C* | • | • | • | • |
| *PDE6D* | • | • | • | • |
| *PDE6G* | • | • | • | • |
| *PDE6H* | • | • | • | • |
| *PHOX2B* | • | • | • | • |
| *PLXNA4* | • | • | • | • |
| *POU4F3* | • | • | • | • |
| *PPT1* | • | • | • | • |
| *PRPH2* | • | • | • | • |
| *RBP4* | • | • | • | • |
| *RBP4B* | • | • | • | • |
| *RCVRN* | • | • | • | • |
| *RDH10* | • | • | • | • |
| *RDH8* | • | • | • | X |
| *REEP6* | • | • | • | • |
| *RGR* | • | • | • | • |
| *RGS9* | • | • | • | • |
| *RGS9BP* | • | • | • | • |
| *RH1* | • | • | • | • |
| *RH2* | • | • | • | • |
| *RPE65* | • | • | • | • |
| *RPGR* | • | • | • | • |
| *RRH* | • | • | • | • |

| | | | | |
|---|---|---|---|---|
| *SAG* | • | • | • | • |
| *SALL1* | • | • | • | • |
| *SEMA3A* | • | • | • | • |
| *SEMA3F* | • | • | • | • |
| *SIX4* | • | • | • | • |
| *SLC1A3* | • | • | • | • |
| *SLC24A1* | • | • | • | • |
| *SLC24A2* | • | • | • | • |
| *SLITRK6* | • | • | • | • |
| *SWS1* | X | • | • | • |
| *SWS2* | • | • | • | • |
| *TFAP2A* | • | • | • | • |
| *THY1* | • | • | • | • |
| *TMEM126A* | • | • | • | • |
| *TRPM1* | • | • | • | • |
| *TULP1* | • | • | • | • |
| *VISININ* | • | • | X | • |
| *VSX1* | • | • | • | • |
| *VSX2* | • | • | • | • |
| *WFS1* | • | • | • | • |

**Supplementary Table 5 Amino acid replacement of visual pigments (*LWS*, *SWS2*, *RH1*, *RH2*) and their effects on the wavelength shift of maximal absorption (Δλ).** Amino acid site numbers are based on the bovine rhodopsin.

| Visual pigment | Amino acid change | Δλ (nm) | Source |
|---|---|---|---|
| ***LWS*** | | | |
| | S164A | -7 | (16) |
| | H181Y | -28 | (16) |
| | Y261F | -8 | (16) |
| | T269A | -15 | (16) |
| | A292S | -27 | (16) |
| | S164A & H181Y | +11 | (16) |
| ***SWS2*** | | | |
| | S91P | +10 | (63) |
| | T93L | -9 | (63) |
| | T93V | -6 | (63) |
| | A94S | +14 | (63) |
| | S127C | +2 | (63) |
| | L207I | -6 | (63) |
| | C211S | +2 | (63) |
| | F261Y | +5 | (63) |
| | A269S | +3 | (63) |
| | A269T | +5 | (63) |
| | S292A | +8 | (63) |
| ***RH1*** | | | |
| | D83N | -6 | (62) |
| | N83D | +2 | (62) |
| | G90S | -13 | (62) |
| | E113D | +7 | (62) |
| | T118A | -16 | (62) |
| | E122Q | -20 | (62) |
| | Q122E | +10 | (62) |

| | | | |
|---|---|---|---|
| | I133F | blue-shift | (16) |
| | A164S | +2 | (62) |
| | F261Y | +10 | (62) |
| | Y261F | -8 | (62) |
| | W265Y | -15 | (62) |
| | A269T | +14 | (62) |
| | A292S | -10 | (62) |
| | S292A | +8 | (62) |
| | Q122E & S292A | +26 | (16) |
| ***RH2*** | | | |
| | F49A & L52M | −4 | (61) |
| | T97A | −8 | (61) |
| | D83N & M86T & T97A | −15 | (61) |
| | Q122E | +13-16 | (61) |
| | E122Q | -10 | (16) |
| | S164A | −1 | (61) |
| | F49S & Q122E & S164A | +15 | (61) |
| | L207M | +6 | (16) |
| | Q122E & L207M | +21 | (16) |
| | S292A | +7 | (61) |
| | Q122E & S292A | +17 | (61) |

**Supplementary Table 6 Recombination analyses of the positively selected genes.** The statistical significance for each of the breakpoints found is shown.

| Genes | BPs | AICc | ΔAICc | Breakpoint(LHS *p-value*, RHS *p-value*) | Significance |
|---|---|---|---|---|---|
| *ABCA4* | 2 | 32348.50 | 4.00 | 2748(0.43040, 0.00040), 4473(0.10400, 0.00040) | N.S,N.S |
| *ARR3* | 2 | 7303.37 | 11.42 | 541(0.00040, 0.00040), 867(0.00040, 0.00040) | ***,*** |
| *CCDC66* | / | / | / | / | no recombination |
| *CLN8* | 1 | 6974.88 | 20.84 | 654(0.02580, 0.00020) | ** |
| *CNGA1* | 1 | 11703.50 | 25.62 | 459(0.00220, 0.01180) | ** |
| *CNGB1* | 3 | 12449.40 | 4.12 | 93(0.00600, 0.20100), 351( 0.24840, 1.00000), 510(0.76200, 0.08460) | N.S,N.S,N.S |
| *CNGB3* | 1 | 9297.42 | 15.36 | 412(0.04540, 0.15760) | N.S |
| *COL2A1* | 3 | 14413.60 | 0.73 | 132(0.06120, 0.35820), 1167(0.20820, 0.00060), 1320(0.00060, 1.00000) | N.S,N.S,N.S |
| *GRK1* | 3 | 8812.24 | 3.23 | 117(1.00000, 0.61440) , 287(1.00000, 0.00060) , 453(0.01080, 1.00000) | N.S,N.S,N.S |
| *GUCA1A* | / | / | / | / | no recombination |
| *GUCY2D* | 2 | 19351.50 | 16.78 | 449(0.14480, 0.09080), 690(0.00560, 0.60480) | N.S,N.S |
| *GUCY2F* | 1 | 23712.70 | 9.24 | 185(0.0002, 0.0934) | * |
| *LWS* | 3 | 7539.23 | 35.81 | 309(0.01440, 0.00060), 552(0.00060, 0.00060), 714(0.00060, 0.00180) | **,***,*** |
| *NXNL2* | 1 | 1576.88 | 2.06 | 240(0.07660, 0.54580) | N.S |
| *PCDH15* | 5 | 37648.60 | 26.58 | 1797(0.30500, 0.00100), 2193( 0.00100, 0.22900), 2541(0.00200, 0.16300) | N.S,N.S,N.S |
| | | | | 3456(1.00000, 0.00100), 4485(1.00000, 0.94100) | N.S, N.S |
| *PDE6B* | / | / | / | / | no recombination |
| *PDE6C* | 1 | 14610.80 | 25.04 | 822(0.00340, 0.41540) | N.S |
| *PDE6H* | / | / | / | / | no recombination |
| *RPGR* | 1 | 9033.37 | 36.56 | 435(0.00020, 0.00560) | *** |
| *SAG* | 1 | 5639.12 | 26.09 | 339(0.00020, 0.05800) | * |
| *SLC24A1* | 2 | 9896.00 | 62.88 | 363(0.80240, 0.00040), 588(0.00040, 0.78600) | N.S,N.S |
| *SWS2* | 3 | 6577.10 | 22.39 | 129(0.05160, 0.00060), 372(0.00060, 0.04620), 599(1.00000, 0.08820) | *,**, N.S |

*P<0.1, **P<0.05,***P<0.01; N.S, not significant; BPs: number of breakpoints in the model; AICc: AIC-c score for the best model with this many BPs; ΔAICc: AIC-c improvement relative to the model with one fewer breakpoint; no recombination, no evidence of recombination was found.

**Supplementary Table 7 Primer pairs used for amplifying exons 1-5 of *RH1* gene.** The annealing temperatures (T) for amplicaiton of the different exons are also shown.

| Exon | Primer (5'-3') | T (℃) | Source |
|---|---|---|---|
| Exon 1 | Rho.1F: MGTGGTCYCCAACAAT | 54.0 | This study |
| | Rho.1R: RGTGYCATTTGYYACTTTC | | This study |
| Exons 2-4 | Rhod.2F: GAAATTGCTCTCTGGTCRCTGGTYGT | 63.3 | (64) |
| | Rhod.4R: AAAGAANGCYGGGATGGTCATGAAGA | | (64) |
| Exons 4-5 | Rho.4F: CACYACCCAGAAGGCAGAGAARGAA | 60.9 | This study |
| | Rho.5R: CTCAGCRAGTSAACAGAAAKGCGGA | | This study |

## References

59 Hunt, D. M., Carvalho, L. S., Cowing, J. A. & Davies, W. L. Evolution and spectral tuning of visual pigments in birds and mammals. *Phil Trans Biol Sci* **364**, 2941-2955 (2009).

60 Palczewski, K. *et al.* Crystal structure of rhodopsin: AG protein-coupled receptor. *Science* **289**, 739-745 (2000).

61 Takenaka, N. & Yokoyama, S. Mechanisms of spectral tuning in the RH2 pigments of Tokay gecko and American chameleon. *Gene* **399**, 26-32 (2007).

62 Yokoyama, S. Evolution of dim-light and color vision pigments. *Annu Rev Genom Hum Genet* **9**, 259-282 (2008).

63 Yokoyama, S. & Tada, T. The spectral tuning in the short wavelength-sensitive type 2 pigments. *Gene* **306**, 91-98 (2003).

64 Kimball, R. T. *et al.* A well-tested set of primers to amplify regions spread across the avian genome. *Mol Phylogenet Evol* **50**, 654-660 (2009).
